# Predicting preeclampsia and related risk factors using data mining approaches: A cross-sectional study

**DOI:** 10.18502/ijrm.v19i11.9911

**Published:** 2021-12-13

**Authors:** Zohreh Manoochehri, Sara Manoochehri, Farzaneh Soltani, Leili Tapak, Majid Sadeghifar

**Affiliations:** ^1^Department of Biostatistics, Student Research Committee, Hamadan University of Medical Sciences, Hamadan, Iran.; ^2^Department of Midwifery, School of Nursing and Midwifery, Hamadan University of Medical Sciences, Hamadan, Iran.; ^3^Modeling of Noncommunicable Disease Research Center, Department of Biostatistics, School of Public Health, Hamadan University of Medical Sciences, Hamadan, Iran.; ^4^Department of Statistics, Faculty of Basic Sciences, Bu-Ali Sina University, Hamadan, Iran.

**Keywords:** Preeclampsia, Random forest, C5.0 decision tree, Support vector machine, Logistic regression.

## Abstract

**Background:**

Preeclampsia is a type of pregnancy hypertension disorder that has adverse effects on both the mother and the fetus. Despite recent advances in the etiology of preeclampsia, no adequate clinical screening tests have been identified to diagnose the disorder.

**Objective:**

We aimed to provide a model based on data mining approaches that can be used as a screening tool to identify patients with this syndrome and also to identify the risk factors associated with it.

**Materials and Methods:**

The data used to perform this cross-sectional study were extracted from the clinical records of 726 mothers with preeclampsia and 726 mothers without preeclampsia who were referred to Fatemieh Hospital in Hamadan City during April 2005–March 2015. In this study, six data mining methods were adopted, including logistic regression, k-nearest neighborhood, C5.0 decision tree, discriminant analysis, random forest, and support vector machine, and their performance was compared using the criteria of accuracy, sensitivity, and specificity.

**Results:**

Underlying condition, age, pregnancy season and the number of pregnancies were the most important risk factors for diagnosing preeclampsia. The accuracy of the models were as follows: logistic regression (0.713), k-nearest neighborhood (0.742), C5.0 decision tree (0.788), discriminant analysis (0.687), random forest (0.758) and support vector machine (0.791).

**Conclusion:**

Among the data mining methods employed in this study, support vector machine was the most accurate in predicting preeclampsia. Therefore, this model can be considered as a screening tool to diagnose this disorder.

## 1. Introduction

Pregnancy blood pressure disorders are one of the most common adverse pregnancy outcomes worldwide (1). One of the most important types of these disorders is preeclampsia (2). Preeclampsia, which usually begins after the 20 ${}^{\mathrm{th}}$ wk of pregnancy, is defined as blood pressure of at least 140/90 mm Hg in two separate stages at least four hr apart, along with proteinuria of at least 0.3 g in the urine collected within 24 hr (3). This syndrome, which affects 5-8% of pregnancies worldwide, is one of the leading causes of maternal and fetal mortality (4-6).

The prevalence of preeclampsia varies in different parts of Iran with reports of 4% in rural areas and 10% in urban areas (7). Preeclampsia can lead to complications such as renal necrosis, pulmonary edema, liver rupture, hemolysis, increased liver enzymes, decreased platelet syndrome, and stroke (8). In addition to the above, preeclampsia is associated with intrauterine fetal growth restriction, bleeding problems, preterm delivery, and low birth weight (9). In addition to threatening the mother's physical health, this disorder can lead to emotional disorders such as anxiety and depression (8). Unfortunately, no simple test is available to diagnose preeclampsia, and diagnosis is performed only by repeated visits during pregnancy, repeated blood pressure measurements, and urine analysis, which are costly and highly sensitive, and delay diagnosing the disorder (3). Therefore, simple alternative diagnostic methods are needed.

One of these prediction methods is classification. The simplest type of classification method divides subjects into two groups such as healthy and sick. Classification is one of the main tasks in the field of data mining. Data mining, which is the science of exploring knowledge from data, identifies potential trends, invisible communications, and hidden patterns between the mass of datasets (10). Data mining methods are known as a useful tool for diagnosing a variety of diseases or predicting clinical consequences. In most studies, these techniques are more accurate than conventional methods of predicting disease (11, 12). So far, various classification methods have been introduced to the field of data mining, the most common of which are logistic regression (LR), k-nearest neighborhood (k-NN), C5.0 decision tree, random forest (RF), support vector machine (SVM) and linear discriminant analysis (LDA) (13, 14). LR, C5.0 decision tree and RF, in addition to predicting disease status, can identify the risk factors related to a disease.

Therefore, in this study, we aimed to select the model with the best performance among the six data mining approaches mentioned above, and to use it as a screening tool to identify mothers with preeclampsia. We also used LR, C5.0 decision tree and RF to identify the risk factors associated with this syndrome. It should be noted that in employing these models, we used clinical data recorded in the hospital which did not require large expenses.

## 2. Materials and Methods

### Study design and participants

In this cross-sectional study, information about 1452 pregnant women who were referred to Fatemieh Hospital in Hamadan City, in western Iran that underwent prenatal care during April 2005–March 2015 was used. As the inclusion criteria, all women should complain about hypertension problems (blood pressure of at least 140/90 mm Hg). Mothers with fetal death or multiple pregnancy (e.g., twins) were excluded from the study. Information about each mother was obtained from their clinical files. This information was extracted using a checklist that included the following variables: age, education, job, number of pregnancies, number of children, sex of the fetus, the season of pregnancy, underlying conditions including hypertension, kidney disease, heart disease and diabetes, and finally preeclampsia status (categorized as with or without preeclampsia).

### Software

After collecting the information, approximately 70% of the total sample (1016 people) were used for training and about 30% of the remaining sample (435 people) were used to test the models. The training data were used to build and train the model and the test data were used to assess the performance of the model to predict healthy or patient classes (in our study–with preeclampsia or without preeclampsia). Data were processed in the R 3.2.2 software environment. To build the models using the R software, the C50 package for C5.0 decision tree, e1071 package for SVM, random Forest package for RF, and MASS package for LDA were used and their performance was compared using accuracy, sensitivity, and specificity criteria on the test data.

### Ethical considerations

Details of the women were collected without including the name. In addition, individuals' information was kept confidential. The study was approved by the Vice-Chancellor for Research and Technology, Hamadan University of Medical Sciences, Hamadan, Iran (Code: 9505122624).

### Statistical analysis

#### Logistic regression (LR)

LR is a standard method for binary classification. In LR, Y represents the binary response variable (in this study, Y = 1 for a subject with preeclampsia and Y = 0 for without preeclampsia) and *X ${}_{1}$ , ..., X ${}_{P}$* represent the vector features (in our study, clinical features of patients). In this case, the probability of Y = 1 (probability of belonging to the class of mothers with preeclampsia) was calculated as follows: 


$$P\left(Y=1\right)=\frac{\exp\;(\beta_{0}+\beta_{1}X_{1}+\cdots +\beta_{P}X_{P})}{1+\exp\;(\beta_{0}+\beta_{1}X_{1}+\cdots +\beta_{P}X_{P})}$$

Based on this, the person would have been assigned to class 1 if P (Y = 1) > C and otherwise to class 0, where C was a fixed number (15, 16).

#### k-nearest neighborhood (k-NN)

The k-NN algorithm is a non-parametric method that is commonly used for classification and regression problems. It is one of the most widely used algorithms due to its simplicity and ease of implementation. In order to classify a new person into one of the healthy or patient classes (in our study - with preeclampsia or without preeclampsia) that displayed in the feature space with a point, k-NN calculates the distance between this point and the other points in the training dataset. Euclidean distance is usually used as the distance criterion. This distance between A and B was calculated as follows: 


 dist A,B=∑i=1m(xi−yi)2m

Then the point was assigned to a class in the k nearest neighborhood where k was an integer (17).

#### Linear discriminant analysis (LDA)

LDA is a classic classifier that uses a linear decision function for classification. In this method, a linear combination of independent variables (features) was used to separate the dependent variable classes in the best way. In other words, the goal was to find a linear function that maximized the probability of separation between the two groups. The conditional probability of independent variables given the label class was used to predict the label class of a new case. A function was used to maximize the distance between the mean of the groups so that the scatter within the classes was minimized and the scatter between classes was maximized (18).

#### Decision tree

The structure of the decision tree is similar to a tree, which includes roots, branches, and leaves. The classification tree divided the data (parent node) into two subsets (children node) using a split criterion. This division continued until we finally reached a homogeneous level of response in each node. In decision tree, the branches represent combinations of input features and the leaves represent the labels of the target class (in our study, 0 was the label of the without preeclampsia class and 1 was the label of the with preeclampsia class) (19).

The rules produced by the decision tree were explained using the logical terms “if" and “then". The decision trees that are most common are ID3, C4.5, C5.0 and CART (16). The C5.0 decision tree, which was introduced by Quinlan in 1987, is modified from the C4.5 version (20). C5.0 decision tree is faster than the C4.5 and produces more precise rules (21). Therefore, in this study, we used this type of decision tree.

#### Random forest (RF)

RF is an “ensemble learning" technique that involves a large number of decision trees whose variance is lower than that of a single decision tree. Each RF tree was based on a bootstrap sample that was randomly extracted from the original dataset and built using the CART method and the Decrease Gini Impurity split criterion (22).

#### Support vector machine (SVM)

SVM was introduced by Vapnik in 1979. Its goal is to find the best function for classification so that the members of the two classes (in our study-with preeclampsia or without preeclampsia) can be distinguished in the dataset. Assuming that the classes are linearly separable, to separate the classes, a hyperplane with a maximum margin is created (23). But in cases where the input dataset is not linearly separable, using the kernel function the data are mapped to the feature space with a high dimension so that they can be separated linearly in this new space. The most common kernel functions used in SVM are linear, polynomial, and radial basis function (RBF) kernel. In this study, we used the RBF kernel because it has more generalizability than other kernel functions (24). The equation we used for RBF kernel was as follows: 


$$k\left(x,x_{i}\right)=\exp\left(\frac{{-∥x-x_{i}∥}^{2}}{2\gamma^{2}}\right),\gamma >0$$

## 3. Results

Of the 1452 pregnant women in this study that underwent prenatal care, 726 subjects were diagnosed with preeclampsia, and 726 subjects were diagnosed without this condition. The mean age for mothers with preeclampsia (35.41 ± 7.91) was higher than the mean age for mothers without preeclampsia (34.53 ± 6.72) (p = 0.02). Most mothers in the preeclampsia group had blood type O (63.4%), while in the group without preeclampsia, the most common blood type was A (58.7%). Most mothers with preeclampsia had a male fetus (68.7%), and this percentage was found to be 58.9% for mothers without the disorder; this difference was statistically significant (p < 0.001).

Most mothers in the preeclampsia group (42.1%) had high blood pressure, and in the control group, the majority did not have any underlying conditions (82.2%). The highest frequency of preeclampsia was observed in March and the lowest in July, and there was a significant relationship between hot and cold months and preeclampsia (p < 0.001). The results of comparing the other variables in the two groups are reported in table I. All variables were entered into the LR model using the stepwise backward elimination method and the odds ratios for each variable are given in table II.

The rules derived from the C5.0 decision tree are stated in table III. Among the variables used in this study, the variables that contributed most to the extracted rules from the C5.0 decision tree were underlying conditions (100%), degree of education (21.44%), pregnancy season (44.55%) and the number of pregnancies (10.42%).

**Figure 1:**
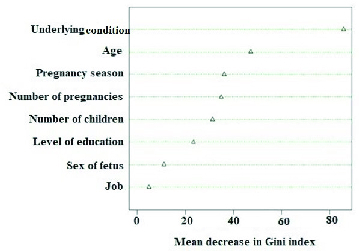
Factors predicting preeclampsia.

Based on the results from the RF model, the most important variable in predicting preeclampsia was the underlying conditions. Age, pregnancy season, number of pregnancies, and the number of children were other important variables in predicting preeclampsia. The order of factors that were important in predicting preeclampsia is reported in figure 1.

For the k-NN model, the highest accuracy was obtained for k = 21, and in the SVM model, the maximum accuracy for the RBF kernel function was obtained for the parameters C = 17034.19, γ = 0.3049167 (24).

To evaluate and compare the performance of the models proposed for the classification of mothers with vs. without preeclampsia, the models were performed on test data (n = 435). The performance evaluation criteria for the models, including accuracy, sensitivity, and specificity, were calculated using the following equation and the results are shown in table IV. 


 Accuracy = TP + TN N

Sensitivity = TP  TP + FN

Specificity = TN  FP + TN

True positive (TP), true negative (TN), false positive (FP), and false negative (FN) were obtained from the classifier (24).

**Table 1:** Demographic features of participants in terms of preeclampsia status


** Variables**		**With preeclampsia **	**Without preeclampsia **	**p-value**
Quantitative features*				
** Age (yr)**		34.53 ± 6.72	35.41 ± 7.91	0.02 ${}^{a}$
** Number of pregnancies**		1.81 ± 1.26	1.29 ± 0.93	< 0.001 ${}^{a}$
** Number of children**		0.67 ± 0.21	0.24 ± 0.15	< 0.001 ${}^{a}$
Qualitative features**				
** Degree of education**				
	**Lower than diploma**	419 (57.7)	318 (43.8)	
	**Diploma**	83 (11.4)	29 (4.0)	
	**Academic**	224 (30.9)	379 (52.2)	< 0.001 ${}^{a}$
** Job**				
	**Housewife**	698 (96.1)	708 (97.5)		
	**Employee**	26 (3.6)	17 (2.4)	
	**Self-employed**	2 (0.3)	1 (0.1)	0.32
** Sex of fetus**				
	**Female**	227 (31.3)	321 (44.2)		
	**Male**	499 (68.7)	405 (55.8)	< 0.001 ${}^{a}$
** Pregnancy season**				
	**Spring**	187 (25.8)	206 (28.4)	
	**Summer**	147 (20.2)	321 (44.2)	
	**Autumn**	188 (25.9)	103 (14.2)	
	**Winter**	204 (28.1)	96 (13.2)	< 0.001 ${}^{a}$
** Underlying condition**				
	**No condition**	293 (40.4)	597 (82.2)		
	**Kidney disease**	11 (1.5)	3 (0.4)		
	**Heart disease**	22 (3.1)	5 (0.7)	
	**Diabetes**	94 (12.9)	21 (2.9)	
	**Hypertension**	306 (42.1)	100 (13.8)	< 0.001 ${}^{a}$
** Blood group**				
	**A**	126 (17.4)	426 (58.7)		
	**B**	101 (13.8)	95 (13.1)		
	**AB**	39 (5.4)	48 (6.6)		
	**O**	460 (63.4)	157 (21.6)	< 0.001 ${}^{a}$
*Data presented as Mean ± SD and Student's *t* test was used. **Data presented as n (%) and Chi-square test was used. ${}^{a}$ P < 0.05				

**Table 2:** LR model with stepwise method


**Variable**	**B**	**OR (95% CI)**	**p-value**
**Constant**	0.346	1.414 (0.636, 3.140)	0.40
**Age (yr)**	0.032	1.032 (1.012, 1.053)	< 0.01*
**Number of pregnancies**	-0.328	0.720 (0.618, 0.839)	< 0.001*
**Sex of fetus **	-0.437	0.646 (0.472, 0.884)	0.01*
**Pregnancy season (summer)**	0.422	1.526 (0.445, 0.967)	0.03*
**Pregnancy season (autumn)**	-0.965	0.381 (0.248, 0.584)	< 0.001*
**Pregnancy season (winter)**	-0.856	0.425 (0.276, 0.654)	< 0.001*
**Underlying condition (diabetes)**	-1.992	0.136 (0.074, 0.251)	< 0.001*
**Underlying condition (kidney disease)**	-2.724	0.066 (0.008, 0.558)	0.01*
**Underlying condition (heart disease)**	-3.384	0.034 (0.004, 0.275)	< 0.001*
**Underlying condition (hypertension)**	-1.533	0.216 (0.153, 0.305)	< 0.001*
**Degree of education (diploma)**	-0.634	0.530 (0.276, 1.021)	0.06*
**Degree of education (academic)**	0.441	1.555 (1.136, 2.127)	0.01*
*P < 0.05, P-value based on score test			

**Table 3:** The rules generated by the C5.0 decision tree


**No.**	**Generated rules**
**1**	If the number of pregnancies > 1, and without underlying condition then the possibility of belonging to the group with preeclampsia is 79.6%
**2**	If the sex of fetus is female, without underlying condition, and pregnancy season is summer, then the possibility of belonging to the group without preeclampsia is 87%
**3**	If number of children > 1, pregnancy season is winter, and with underlying condition, then the possibility of belonging to the group with preeclampsia is 86%
**4**	If the number of pregnancies > 1, with degree of education lower than diploma, and with underlying condition, then the possibility of belonging to the group with preeclampsia is 86%
**5**	If the age < 31 yr, with academic degree of education, and without underlying condition, then the possibility of belonging to the group without preeclampsia is 83%

**Table 4:** The results of the assessment classifier models


**Model**	**Accuracy**	**Sensitivity**	**Specificity**
**LR**	0.713	0.649	0.797
**KNN**	0.742	0.729	0.754
**C5.0**	0.788	0.736	0.846
**DA**	0.687	0.477	0.891
**RF**	0.758	0.737	0.776
**SVM**	0.791	0.800	0.780
LR: Logistic regression, KNN: K-nearest neighborhood, C5.0: C5.0 decision tree, DA: Discriminant analysis, RF: Random forest, SVM: Support vector machine			

## 4. Discussion

Due to the serious risks that preeclampsia poses to the mother and fetus, it is important to use methods that can predict this outcome. However, despite recent advances in the etiology of preeclampsia, to date, no clinical screening tests have been identified to diagnose the disorder (25). Identifying the underlying and predictive factors of preeclampsia can play a significant role in reducing mortality and complications in the mother and fetus. In addition to identifying the risk factors associated with preeclampsia, this study aimed to compare common data mining approaches and select the strongest model to help professionals in this field. In this section, we will first consider the most important risk factors associated with preeclampsia and then discuss the performance of the data mining models.

According to the results of the univariate analysis (Table I), most mothers in the preeclampsia group had blood type O (63.4%). However, in the group without preeclampsia, most people had blood type A (58.7%). A study by Elmugabil also showed that mothers with blood type O were more at risk for eclampsia (26). The distribution of the sex of the fetus in the two groups was also significantly different (p < 0.001) so that most mothers in the preeclampsia group had a male fetus (68.7%). Among the models adopted in the present study, LR, C5.0 decision tree, and RF models, in addition to predicting the response variable (preeclampsia status), also identified the risk factors related to the condition. Based on the results of fitting these three models, the variables of underlying condition, age, pregnancy season, and number of pregnancies were the most important risk factors in diagnosing preeclampsia. In a study by Rezende and co-authors, a significant difference was obtained between the preeclampsia and control groups in terms of variables such as gestational age, chronic hypertension, and type 1 and type 2 diabetes, which is consistent with the results of the present study (27). In a study conducted by Farzaneh and co-workers to identify risk factors for preeclampsia, a history of hypertension was one of the main risk factors, but contrary to our study, there was no association between preeclampsia and age, number of pregnancies, or gestational age (28).

Based on the results of fitting the data mining models, the accuracy with the test data was as follows: SVM (0.791), C5.0 (0.788), RF (0.758), k-NN (0.742), LR (0.0713), LDA (0.687). Therefore, according to the obtained results, SVM had the highest accuracy among the fitted models. In a study conducted by Asfaw to predict diabetes, the SVM model had the highest accuracy among the six data mining models that were used, which included SVM, decision tree, RF, Naïve Bayes, LR, and k-NN (29). But this result is inconsistent with a study conducted by Basu et al. to diagnose breast cancer where RF had the highest accuracy (92.98%) among the classification methods decision tree, SVM, k-NN, and RF; SVM was in second place with an accuracy of 61.403%, and k-NN had the lowest accuracy (30). In fact, in most data mining studies, there is close competition between the SVM and RF models so that in some studies, SVM accuracy exceeds RF (29), and in other cases, the opposite is true (30). In the present study, after SVM, C5.0 decision tree and RF also performed well. One of the advantages of C5.0 decision tree and RF was that these models, in addition to predicting with acceptable accuracy, were also able to identify risk factors affecting the condition whereas this advantage did not exist for SVM, k-NN, and LDA. Another advantage of the decision tree model was that it provided an intuitive image of the impact of risk factors by presenting a series of rules.

In our study, the LDA model had the least predictive accuracy, which is inconsistent with the results of a study conducted by Maroco and colleagues to predict dementia by comparing artificial neural network, SVM, RF, LR, and decision tree models; in this study, LDA was the most accurate model after SVM and RF (31). In our study, after LDA, the LR model had the lowest ability to diagnose patients with preeclampsia (accuracy = 0.713). This is inconsistent with the results of a study in which the LR model had the highest diagnostic power in predicting breast cancer compared to models such as Naïve Bayes, k-NN, Ada Boost, and decision tree-J 48 (32). However, the LR model had the advantage that, by interpreting the coefficients in this model and also calculating the value of the odds ratios, the effect of each variable on the response variable (preeclampsia) could be calculated and interpreted. This advantage did not exist in the other classification methods. For example, for the degree of education variable, an odds ratio of 1.55 was obtained (Table II). This means that having an academic education compared with education less than diploma increased the chances of developing preeclampsia by 0.55.

One of the limitations of the present study was the potential recall bias, because pregnant mothers may not remember much of the information about pregnancy; this is an unavoidable error in data collection in such studies. Finally, it is recommended that these models be used on more genetic and clinical risk factors to achieve higher diagnostic power.

## 5. Conclusion

Among the data mining models employed in this study, the SVM model had the highest prediction accuracy. Therefore, we can conclude that this model can be used as a screening tool to help predict preeclampsia. Based on the results of the RF model, which also showed good performance in this study, the variables of underlying condition, degree of education, pregnancy season, and the number of pregnancies were the most important risk factors associated with preeclampsia. Therefore, by controlling these factors and also regularly monitoring the blood pressure of mothers with these risk factors, the potential risks associated with this syndrome can be reduced.

##  Conflict of Interest 

The authors declare that they have no conflict of interest.
